# Study on Influence of Rare Earth Ce on Micro and Macro Properties of U75V Steel

**DOI:** 10.3390/ma17030579

**Published:** 2024-01-25

**Authors:** Guangqian Feng, Lei Ren, Jichun Yang

**Affiliations:** School of Materials and Metallurgy, Inner Mongolia University of Science and Technology (IMUST), Baotou 014010, China; 15379229582@163.com (G.F.); yangjichun1963@163.com (J.Y.)

**Keywords:** U75V steel, rare earth Ce, inclusions, mechanical property

## Abstract

Non-metallic inclusions in steel have great influence on the continuity of the steel matrix and the mechanical properties of steel. The precipitation sequence of Ce inclusions in molten steel is predicted by thermodynamic calculations. The results show that Ce content will affect the precipitation sequence of rare earth inclusions in molten steel, and the formation of CeO_2_, Ce_2_O_3_ and CeAlO_3_ will be inhibited with the increase in Ce content. Our laboratory smelted the test steel without rare earth additive and the test steel with rare earth Ce additive (0.0008%, 0.0013%, 0.0032%, 0.0042%). It was found that the MnS inclusions and inclusions containing Al, Ca, Mg and Si oxides or sulfides in the steel after rare earth addition were modified into complex inclusions containing CeAlO_3_ and Ce_2_O_2_S. The size of inclusion in steel was reduced and the aspect ratio of inclusion was improved. The addition of Ce also improved the grain size of U75V steel and significantly refined the pearlite lamellar spacing. After mechanical property testing of the test steel, it was found that when Ce is increased within 0.0042%, the tensile and impact properties of U75V steel are also improved.

## 1. Introduction

As an important component of railway tracks, heavy rail steel needs to withstand the huge pressure, impact and wear of the wheel, so more attention should be paid to its mechanical properties whilst ensuring the output of heavy rail steel [1,2]. As one of the important factors that reduces the mechanical properties of steel, the quantity, size, distribution and chemical composition of nonmetallic inclusions seriously affect the continuity of the steel matrix and lead to the decline of its mechanical properties [3,4,5,6,7]. In particular, plastic MnS inclusions easily grow into large-size long strip inclusions during the solidification process of liquid steel; this is the source of steel crack initiation [8]. During hot rolling, MnS inclusions deform and extend along the rolling direction, which increases the anisotropy of steel and seriously affects the tensile, impact, ductility and other properties of steel [9,10,11,12]. Improving the non-metallic inclusions in steel can effectively improve the mechanical properties of steel, and some studies have found that rare earth treatment can effectively improve the inclusions in steel.

Because of its unique electron layer structure, rare earth Ce is widely used for modifying inclusions in steel, improving solidification structure, microalloying and so on [13,14,15,16,17,18,19]. Rare earth elements have a strong affinity with harmful elements such as O and S in steel [20,21]. When rare earth Ce is added to steel, high-melting-point rare earth inclusions such as CeAlO_3_ and Ce_2_O_2_S will be generated first, which can effectively modify oxides or sulfides in steel. Such rare earth inclusions have the characteristics of small size, regular shape and dispersion [22], and they have little impact on the continuity of the steel matrix [23]. In addition, the thermal expansion coefficient of rare earth inclusions is closer to that of the steel matrix, which can effectively improve the mechanical properties of steel [24]. Z. Adabavazeh et al. [25] found that rare earth inclusions such as Ce_2_O_2_S and CeAlO_3_ appeared in Commercial SS400 steel after a small amount of rare earth Ce was added. With the increase in rare earth Ce content, the CeAlO_3_ inclusions disappeared, and most of the steel was rare earth oxides and rare earth oxygen sulfides. After adding a small amount of rare earth, the small and medium size inclusions in Commercial SS400 steel increase and the large-size inclusions decrease. However, with the increase in Ce content, the inclusion size increases again due to the collision and aggregation of inclusions. Zhao Qingbo et al. [26] found that the addition of rare earth Ce could refine the grain of medium manganese steel and improve its comprehensive properties. Previous studies have found that the addition of rare earth Ce has a positive effect on a variety of steels, such as IF steel and medium manganese steel. As far as U75V steel is concerned, long irregular MnS inclusions in steel cause considerable harm to the mechanical properties of U75V steel. The use of rare earth treatment can reduce the activity of S in the molten steel, affect the combination of Mn and S and reduce the formation of pure MnS inclusions under supersaturated conditions. However, the addition of excessive rare earth will lead to a sharp increase in the number of rare earth inclusions, and the collision and aggregation in the molten steel will lead to the formation of large-size inclusions. Therefore, the content of rare earth should be attended to when treating the inclusions in the rare earth steel.

Taking U75V heavy rail steel as the experimental object, this study studies the influence of Ce content on the composition, size, distribution and microstructure of MnS and other non-metallic inclusions in U75V heavy rail steel, and it explores the changes in the mechanical properties of U75V steel before and after adding rare earth Ce. It is expected that this study can provide some reference for the application of rare earth in heavy rail steel.

## 2. Experimental Method

### 2.1. Preparation of Experimental Steel

**Figure 1:**
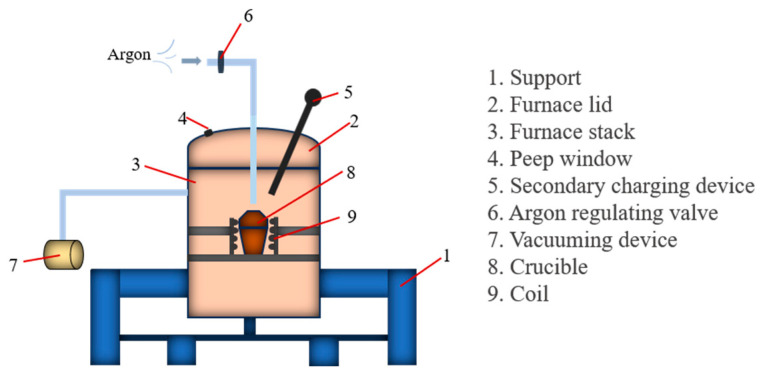
Structure diagram of vacuum induction furnace.

The experimental U75V steel was smelted in a ZG-0.02 20 Kg vacuum induction furnace; the structure diagram is shown in Figure 1, and the raw material composition of the experimental steel is shown in Table 1. The smelting steps of the experimental steel are as follows: (1) The iron rod required for smelting the test steel is placed into the crucible, and the rare earth Ce, ferrosilicon, ferromanganese and ferrovanadium alloys are placed into the secondary charging device. (2) In order to eliminate the impact of impurities in the air, the vacuum furnace is vacuumed first and then filled with high purity argon. (3) After aeration, heating is carried out; the initial power is 5 Kw and then increased at a rate of 3 Kw/20 min until the iron rod is completely melted. (4) After the iron rod has been completely melted for 5 min, the secondary feeding is carried out, and the rare earth and alloy are added. After waiting for about 10 min, the rare earth and alloy are fully melted and evenly mixed with the liquid steel, the heating device is turned off and the steel is produced after the ingot is cooled. (5) In order to eliminate the uneven stress of casting, we retain the dense part of the ingot and roll it.

**Figure 2:**
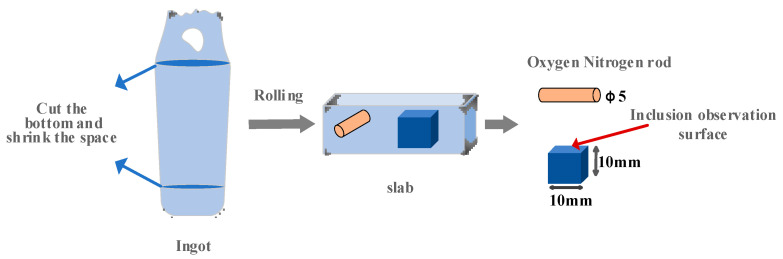
Sampling and processing diagram of ingot.

The O content of the test steel was measured using an ON-3000 oxygen and nitrogen analyzer (Ncs Testing Technology Co., Ltd., Beijing, China), the Ce content of rare earth was determined by an ion spectroscopy–mass spectrometer (ICP-MS), and the other components were detected using a Labspark1000 direct reading spectrometer (Ncs Testing Technology Co., Ltd., Beijing, China). The composition of the experimental steel is shown in Table 2; it conforms to the national standard and has certain reliability. The sampling and processing diagram of the sample is shown in Figure 2. The processed sample was polished until the surface was smooth and without scratches, and the sample was observed by a FEI-QUANTA400 scanning electron microscope (FEI, Hillsboro, OR, USA) and analyzed by its energy spectrum.

### 2.2. Thermodynamic Calculation

The Wagner model based on the change of Gibbs free energy is used to calculate the formation of Ce inclusion; the components used in the calculation are shown in Table 3. According to the composition of U75V steel in Table 3, the thermodynamic relationship between O, S, Al and Ce in liquid steel is discussed, so as to explore the possibility of Ce inclusion formation. In this calculation, 1 mol of cerium is taken as the standard, and the Gibbs free energy of each reaction of U75V steel at different temperatures is calculated according to the above model. The possible chemical reactions and standard Gibbs free energy are shown in Table 4 [27,28]. The interaction coefficients of various elements in liquid steel at 1600 °C are shown in Table 5 [20,27,29]. The interaction coefficients of each element in liquid steel at 1650 °C and 1550 °C are deduced by using a quasi-normal solution model. The theoretical values calculated by this model are close to the experimental values and within the allowable error range of thermodynamic calculation. The calculation method is shown in Equation (1). In order to ensure the full reaction of Ce-containing oxygen sulfide and sulfide in molten steel, the ratio of oxygen to sulfur in the calculation for Ce-containing inclusions is 0.1 < O/S < 0.2.

The Gibbs free energy of each reaction, as generated by rare earth inclusions in U75V steel under different rare earth contents and presented in Table 4 and Table 5, is calculated according to the combination of Equations (1)–(4):

(1) $$e_{i\;(T)}^{j}=(\frac{2538}{T}-0.355)e_{i\;(1873)}^{j}$$

(2) $$\mathrm{lg}f_{i}=\sum_{j=1}^{n}e_{i}^{j}w[j]$$

(3) $$a_{i}=f_{i}w[i]$$

(4) $$\Delta G=\Delta G^{\theta }+RT\ln K$$

where $e_{i\;(T)}^{j}$ is the interaction coefficient of element j on i at T(K) temperature, and $f_{i}$ is the activity coefficient of element i; $e_{i}^{j}$ is the interaction coefficient of element j on i at 1600 °C; w[i] (wt%) is the mass fraction of element i; $a_{i}$ is the activity of element i, and the activity product of pure matter is 1; R is the gas constant (J·mol^−1^·K^−1^), where K is the ratio of the product activity product to the reactant activity product.

## 3. Results and Discussion

### 3.1. Thermodynamic Calculation of Ce-Containing Inclusions

#### 3.1.1. Precipitation Sequence of Ce-Containing Inclusions at 1600 °C

**Figure 3:**
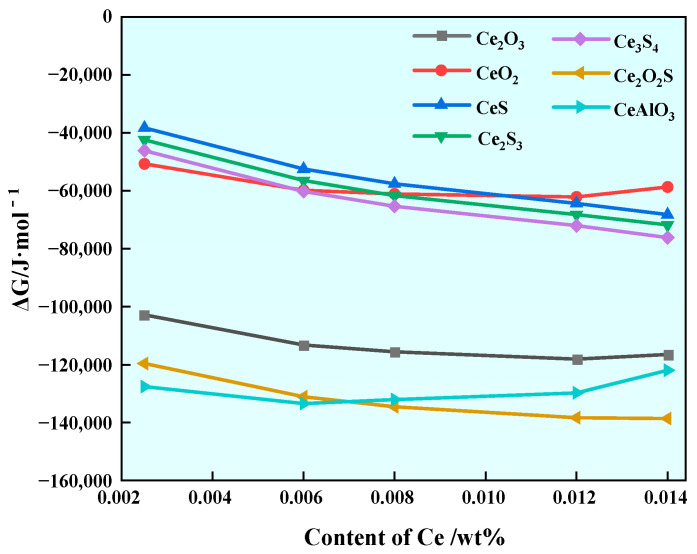
Gibbs free energy generated by Ce inclusion in steel with different Ce contents at 1600 °C.

In order to predict the formation of rare earth inclusions in steel, the Wagner model was used to calculate the Gibbs free energy of the reaction of various rare earth inclusions in steel with different Ce contents at 1600 °C, and the calculation results are shown in Figure 3. It can be seen that the ΔG of each reaction is less than zero at 1600 °C, indicating that under the above smelting conditions, each reaction in Table 4 can spontaneously proceed. In addition, with the increase in Ce content, the Gibbs free energy of CeO_2_ and CeAlO_3_ decreases first and then increases. Under the condition of a certain Al content, when Ce < 0.0075%, the Gibbs freedom of CeAlO_3_ is the lowest, and the inclusion of CeAlO_3_ will be first formed in the molten steel. When Ce > 0.0075%, the Gibbs freedom of Ce_2_O_2_S is the lowest, and the inclusion of Ce_2_O_2_S is first formed in the molten steel. The sequence of precipitation of Ce inclusions at 1600 °C is shown in Table 6. It has been determined that U75V steel mainly contains MnS inclusions and Al_2_O_3_ inclusions [30]. After rare earth Ce is added at steelmaking temperature, Ce will first form Ce_2_O_2_S and CeAlO_3_ containing other Ce inclusions with O, S, Al and other elements in the steel. With the decrease in temperature, there may be Ce inclusions and Ca, Al and Si complex inclusions in the steel; such inclusions have a regular appearance and small size, and the addition of rare earth Ce can play a certain role in modifying the inclusions in U75V steel.

#### 3.1.2. Effect of Temperature and Ce Content on the Formation of Ce-Containing Inclusions

**Figure 4:**
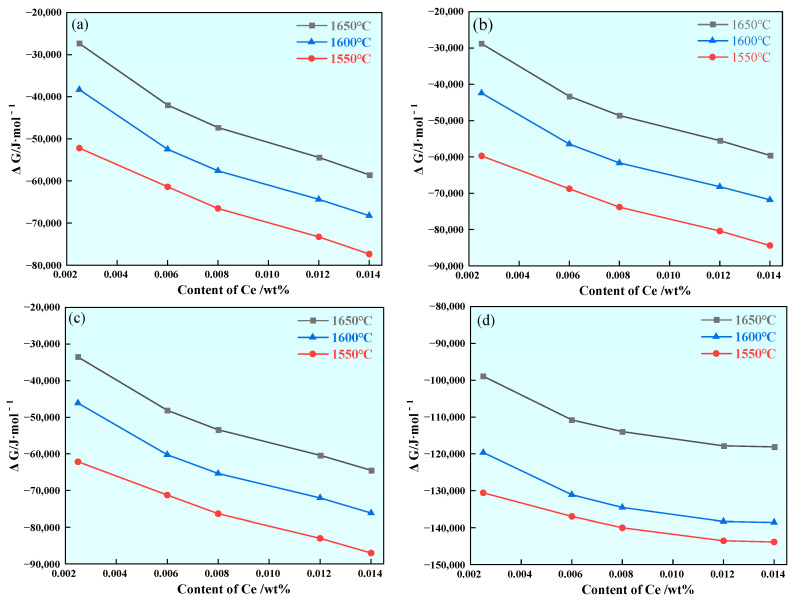
Effect of changes in Ce content on ΔG generated by CeS, Ce_2_S_3_, Ce_3_S_4_ and Ce_2_O_2_S inclusions during cooling: (**a**) CeS, (**b**) Ce_2_S_3_, (**c**) Ce_3_S_4_and (**d**) Ce_2_O_2_S.

In order to investigate the stability of Ce inclusions during temperature reduction, the Gibbs free energies of the reactions at 1650 °C, 1600 °C and 1550 °C for the formation of rare earth inclusions were calculated. The results show that the ΔG of the reaction between Ce-containing oxygen sulfide and Ce-containing sulfide decreases with the increase in the Ce range from 0.0025% to 0.014%, and the ΔG of the formation of such rare earth inclusions decreases with the decrease in temperature. The results show that CeS, Ce_2_S_3_, Ce_3_S_4_ and Ce_2_O_2_S inclusions can exist stably in steel under an increase in Ce content and decrease in temperature, and the calculation results are shown in Figure 4.

**Figure 5:**
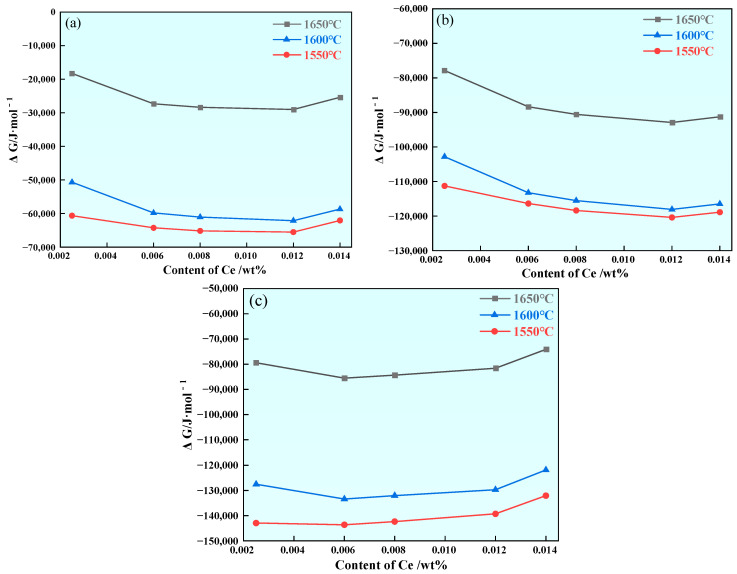
Effects of changes in Ce content on ΔG generated by CeO_2_, Ce_2_O_3_ and CeAlO_3_ inclusions during cooling: (**a**) CeO_2_, (**b**) Ce_2_O_3_ and (**c**) CeAlO_3_.

As the range of Ce increases from 0.0025% to 0.014%, the ΔG generated by CeO_2_, Ce_2_O_3_ and CeAlO_3_ inclusions in U75V steel decreases first and then increases, indicating that excessive Ce will inhibit the formation of these three types of rare earth inclusions as the temperature drops. This indicates that the decrease in temperature does not affect the formation of CeO_2_, Ce_2_O_3_ and CeAlO_3_ inclusions, and the calculation results are shown in Figure 5.

As the most common inclusion in U75V steel, MnS inclusions seriously affect the continuity and mechanical properties of the steel matrix. This calculation aims to explore the modification of MnS inclusions by rare earth, so the S content is set to be high in calculation. After the addition of rare earth Ce, it will first react with O, S, Al and other elements in the steel to form Ce_2_O_2_S and CeAlO_3_ inclusions. With the increase in Ce content, Ce and S will further react to produce rare earth sulfide, so CeS, Ce_2_S_3_, Ce_3_S_4_ and Ce_2_O_2_S inclusions will statically exist in the steel when the Ce content increases, as shown in Figure 4. Due to a series of deoxidation processes before smelting U75V steel, the O content in the steel is low. The Ce_2_O_3_ inclusions generated in steel further react with S to form Ce_2_O_2_S inclusions; the reaction process is shown in Equation (5). With the progress of the reaction, the unstable CeAlO_3_ in steel reacts with Ce and S elements, and the reaction process is shown in Equation (6) [28].

(5) $$Ce_{2}O_{3}(s)+[S]=Ce_{2}O_{2}S(s)+[O]\;\;\Delta G^{\theta }=77360-28.48T$$

(6) $$CeAlO_{3}+[Ce]+[S]=Ce_{2}O_{2}S+[O]+[Al]\;\;\Delta G^{\theta }=12870-32T$$

According to Equations (1)–(6), it can be calculated that at 1600 °C, when the Ce content is 0.0025%, the ΔG of Equations (5) and (6) are −35,001 J/mol and −112,001 J/mol, respectively. When the Ce content is 0.014%, the ΔG of Equations (5) and (6) is −45,512 J/mol and −154,513 J/mol, respectively. The calculation results show that CeAlO_3_ and Ce_2_O_3_ inclusions tend to be unstable with the increase of Ce in the range of 0.0025–0.014%, as shown in Figure 5b,c. Since the reaction of CeO_2_ in molten steel is uncertain, it is further analyzed here.

### 3.2. Inclusions in Steel

#### 3.2.1. Morphology and Composition of Inclusions

**Figure 6:**
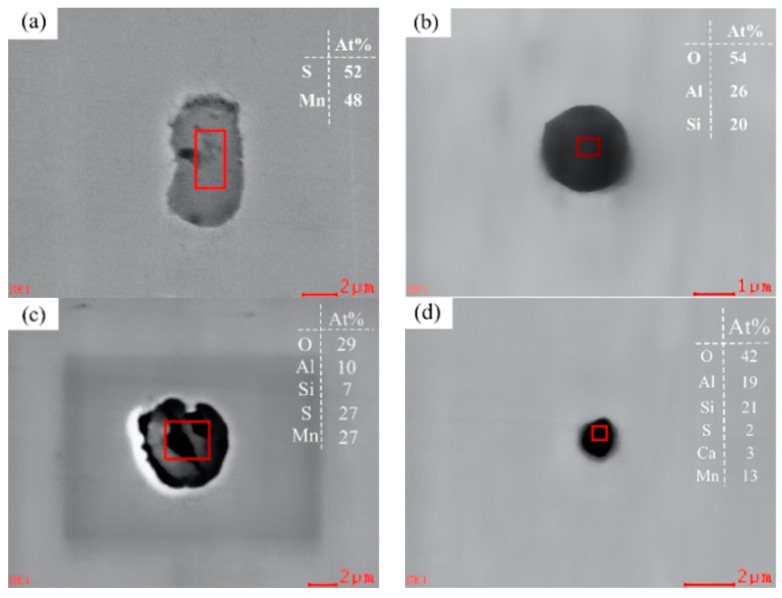
Inclusion species and morphology in Sample S1: (**a**) MnS, (**b**) Si-Al-O, (**c**) Al-Si-O + MnS and (**d**) Al-Si-Ca-Mn-S-O.

In Sample S1 without rare earth additive, the main inclusions were large and irregular pure MnS (Figure 6a) and complex inclusions containing Al, Si, Ca, Mn, S, O and other elements, as shown in Figure 6b–d. Such MnS inclusions are distributed in the steel structure, just as there are many very low strength voids in the steel, and the MnS inclusions will be significantly extended after rolling. In the process of steel use, when subjected to external force, the gap formed by the extension of inclusions will increase with the deformation of the steel matrix, thereby forming cracks in the steel or producing anisotropy of the material properties, thereby shortening the service life of the steel and causing premature failure of the steel [31]. In addition to pure MnS, a part of MnS is deposited on the surface of the oxide or sulfide inclusions to form irregular composite inclusions, as shown in Figure 6c.

**Figure 7:**
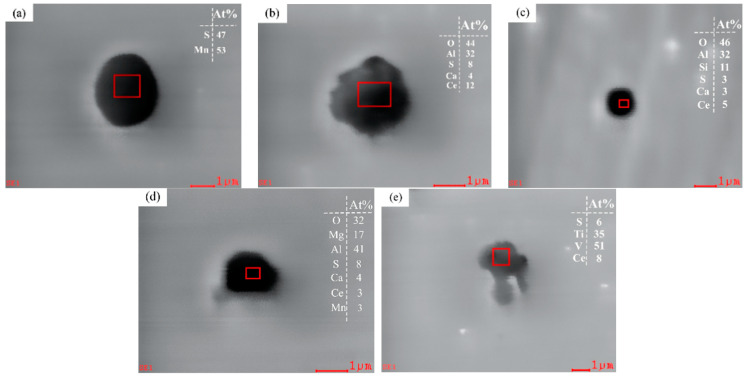
Inclusion in U75V after the addition of rare earth: (**a**) MnS, (**b**) Al-Ca-S-O-CeAlO_3_, (**c**) Al-Si-Ca-S-O-CeAlO_3_, (**d**) Mg-Al-Ca-Mn-O-S-CeAlO_3_, and (**e**) S-Ti-V-Ce.

Compared with the samples without rare earth Ce, most of the pure MnS inclusions in Samples S2–S5 with rare earth Ce added to the steel changed from large and irregular to spherical, as shown in Figure 7a. In addition, rare earth complex inclusions containing Ce, Mg, Al, Si, Ca, O, S and other elements appeared in the samples with rare earth additives. According to the thermodynamic calculation results in Figure 3, CeAlO_3_ inclusions will first be formed in steel when the Ce content is low, as shown in Figure 7b–d. Compared with pure MnS inclusions, these three types of rare earth complex inclusions have a higher melting point, are not easy to deform during hot rolling, have good adaptability to the steel matrix, have a good shape and distribution, reduce the stress concentration and promote the improvement of steel properties. In addition, S-Ti-V-Ce inclusions also appear in the steel, as shown in Figure 7e. The shape of rare earth inclusions with high V and Ti content is irregular. Pure (Ti, V) inclusions have high hardness and do not change their appearance with the change of the steel matrix; thus, they are non-deformable inclusions, which easily produce micro-cracks in the steel after rolling [32]. After rare earth Ce modification treatment, the integration degree of such inclusions with the steel matrix can be enhanced, and the stress concentration and micro-cracks in the steel can be reduced. After the modification of rare earth Ce, the integration degree of such inclusions with the steel matrix can be enhanced, and the stress concentration and micro-cracks of the steel can be reduced. It is worth noting that in addition to the inclusion containing V and Ti, the shape of pure MnS and other rare earth inclusions is relatively regular, all are spherical or spheroid, and the ductility is low in the rolling process, which has little influence on the comprehensive properties of steel [21].

**Figure 8:**
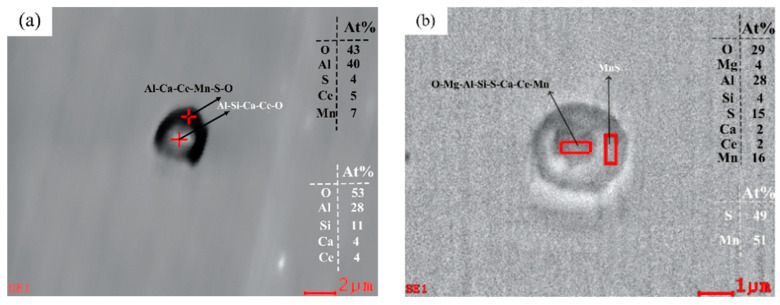

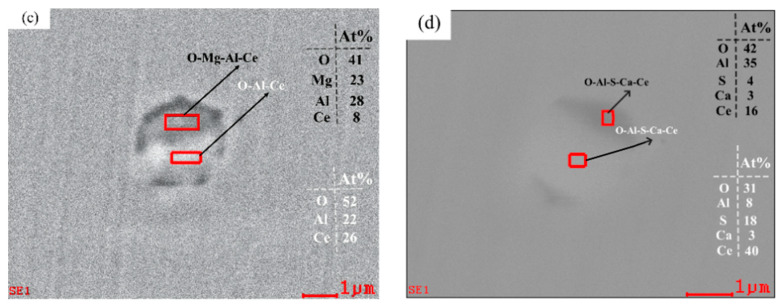
Compound inclusions in U75V after the addition of rare earths: (**a**) Al-Ca-Mn-S-O-CeAlO_3_ + Al-Si-Ca-O-CeAlO_3_ inclusions in S3, (**b**) MnS + Mg-Al-Si-Ca-Mn-O-S-CeAlO_3_ inclusions in S4, (**c**) MgO-CeAlO_3_ + CeAlO_3_ inclusions in S5and (**d**) Al-Ca-S-O-CeAlO_3_ + Al-Ca-O-S-Ce_2_O_2_S inclusions in S5.

In addition, double-layer complex inclusions containing Ce were found in Sample S3 with rare earth additives, as shown in Figure 8a. Double-layer complex inclusions containing Ce inclusions were found in Sample S4, as shown in Figure 8b. These two types of double-layer inclusions have a regular morphology and smaller size than those without the rare earth additive. With the increase in Ce content, complex inclusions containing CeAlO_3_ wrapped by MgO-CeAlO_3_ were found in Sample S5, as shown in Figure 8c. It is noteworthy that Al-Ca-Ce-O-S double-layered composite inclusions were found in Sample S5, as shown in Figure 8d. According to the calculation results of Figure 5c and equation (6), it is not difficult to find that with the increase in Ce content, some CeAlO_3_ inclusions will react with S, O and other elements to form Ce_2_O_2_S inclusions [25], and according to the energy spectrum atom percentage in Figure 8d, it can be deduced that the core part of the inclusion is a complex inclusion containing Ce_2_O_2_S. The low Ce content may be the reason for the absence of Ce_2_O_2_S inclusions in Samples S2–S4.

#### 3.2.2. Changes of Inclusion Species in Steel

**Figure 9:**
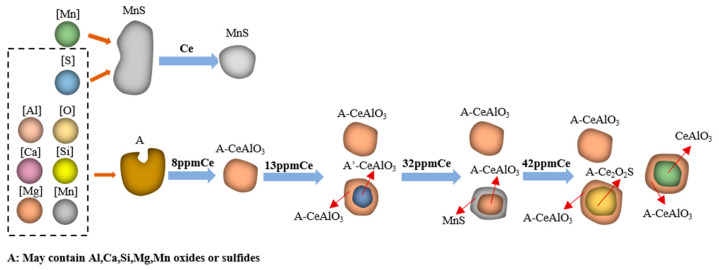
Change diagram of inclusions in U75V steel after adding rare earth Ce.

With the increase in Ce content, the change in inclusions in U75V steel is as shown in Figure 9. From the figure, we can see that there are MnS inclusions with irregular morphologies and compound inclusions containing Al, Si, Ca, Mn, S, O and other elements in steel without rare earth additive. The inclusion containing CeAlO_3_ is formed first after rare earth is added into the steel, and the shape of pure MnS in Samples S2–S5 containing Ce changes from irregular to more regular and spherical. When the Ce content increased to 0.0032%, the double-layer structure inclusions containing CeAlO_3_ inclusions appeared in the steel, and when the Ce content further increased to 0.0042%, the composite inclusions containing Ce_2_O_2_S began to appear in the steel. We found that most of the inclusions in the samples containing rare earth Ce existed in spherical or sphere-like shapes. It is worth noting that when the rare earth content in the steel is less than 0.0042%, MnS inclusions do not disappear, but MnS and complex inclusions containing rare earth elements appear, and the melting point of Ce_2_O_2_S inclusions is higher than that of MnS, so they precipitate before MnS in the solidification process. The activity of S in the molten steel is reduced to some extent, the combination of Mn and S is affected, and the formation of pure MnS inclusions under supersaturation is reduced.

### 3.3. Statistics of Inclusions Size and Aspect Ratio in Steel

#### 3.3.1. Average Inclusion Size and Aspect Ratio

**Figure 10:**
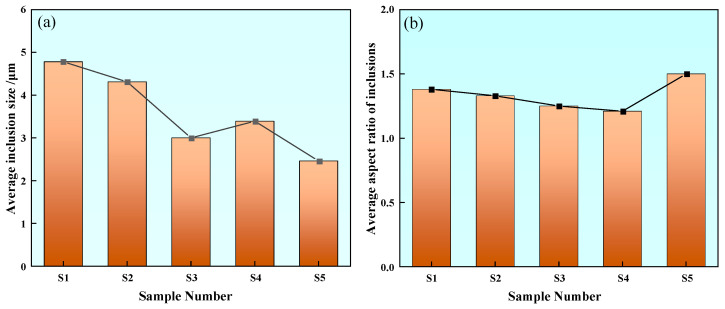
Average inclusion size and average aspect ratio: (**a**) average inclusion size and (**b**) average aspect ratio of inclusions.

In order to quantitatively characterize inclusions in steel, the average size and aspect ratio of inclusions in different samples were calculated. The statistics of average size changes of inclusions are shown in Figure 10a. The average size of inclusions in Sample S1 is 4.78 μm. The average size of inclusions in S2 is 4.31 μm, which is about 10% lower than that in Sample S1. The average size of inclusions in Sample S3 is 3 μm, which is about 37% lower than that in Sample S1. The average size of inclusions in Sample S4 is 3.39 μm, which is about 30% less than that in Sample S1. The average inclusion size of Sample S5 is reduced to 2.46 μm, which is about 49% less than that of Sample S1. The comparison of inclusions in Samples S1–S5 shows that with the increase in rare earth content, the average size of inclusions in steel decreases by 10% to 49%, and the average size of inclusions in Sample S5 containing 0.0042% rare earth Ce is the smallest.

The statistics of the average aspect ratio of inclusions in different samples are shown in Figure 10b. The average aspect ratio of inclusions in Sample S1 is 1.38. The average aspect ratio of inclusions in Sample S2 is 1.33, which is about 3.6% lower than that in Sample S1. The average aspect ratio of inclusions in Sample S3 is 1.25, which is about 9.5% lower than that in Sample S1. The average aspect ratio of inclusions in Sample S4 is 1.21, which is about 12.3% lower than that in Sample S1. The average aspect ratio of inclusions in Sample S5 is 1.5, which is increased by 0.12 compared with Sample S1. The average aspect ratio of inclusions in Samples S1–S5 was compared and it was found that the average aspect ratio of inclusions in steel first decreased and then increased with the increase of rare earth content, and the aspect ratio of Sample S4 was closest to 1. It also shows that the addition of rare earth Ce not only changes the composition of inclusions but also refine the size of inclusions in steel and improve the appearance of inclusions.

#### 3.3.2. Inclusion Size Distribution

**Figure 11:**
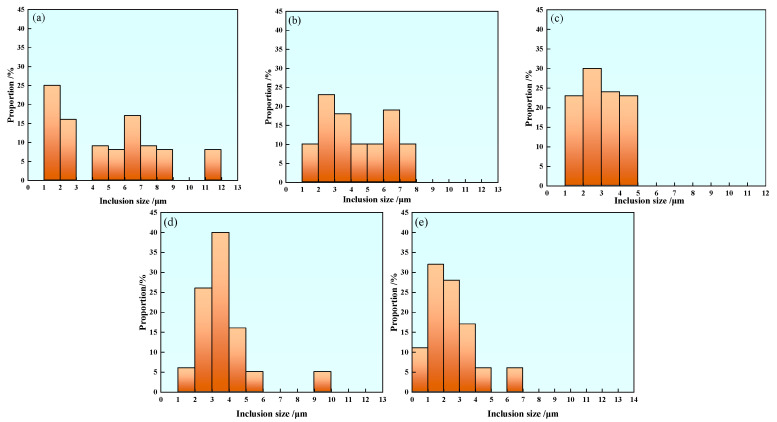
Size distribution of inclusions in U75V steel: (**a**) S1, (**b**) S2, (**c**) S3, (**d**) S4 and (**e**) S5.

Figure 11 shows the average size distribution of inclusions in samples with different rare earth Ce contents. In Sample S1 without rare earth treatment, the inclusion size distribution ranges from 1 to 12 μm, and the size distribution range is large. The inclusion size range of 1–2 μm accounts for about 25%, and the inclusion size range of less than 5 μm accounts for 51%, as shown in Figure 11a. The average size and proportion of inclusions in Sample S2 are shown in Figure 11b. The size of inclusions in Sample S2 containing 0.0008% rare earth Ce is distributed within 1–8 μm, and the proportion of inclusions in the range 2–3 μm is about 23%, and inclusions above 10 μm disappear. The size distribution of inclusions in Sample S3 is shown in Figure 11c. It can be seen that the size of inclusions in Sample S3 is basically less than 5 μm, among which the inclusions with a size of 2–3 μm occupy the highest proportion. The inclusions in Samples S4 and S5 were mainly distributed in the range of 1–6 μm, and the proportion of inclusions smaller than 5 μm was 92%, which was significantly higher than that of Sample S1, as shown in Figure 11d,e. With the increase of rare earth content, the effect of rare-earth-refining inclusions becomes more obvious. The modification of non-metallic inclusions such as MnS in steel by rare earth elements is the main reason for the size refinement of inclusions, but there are still a small amount of large-size inclusions in the samples containing rare earth. The reason may be that the lower rare earth content fails to completely modify the inclusions such as MnS and Al_2_O_3_ in steel, so there is still the possibility of large-size inclusions in steel.

However, excess rare earth will enhance the ability of rare earth elements to combine with other inclusions and generate a large number of rare earth inclusions—resulting in an increased probability of collision and the aggregation of inclusions—and further increase the size of inclusions. In this paper, the proportion of small and medium-sized inclusions in other samples containing rare earth elements is significantly higher than that in Sample S1 without rare earth element additives.

#### 3.3.3. Statistics of Average Size and Aspect Ratio of MnS Inclusions

**Figure 12:**
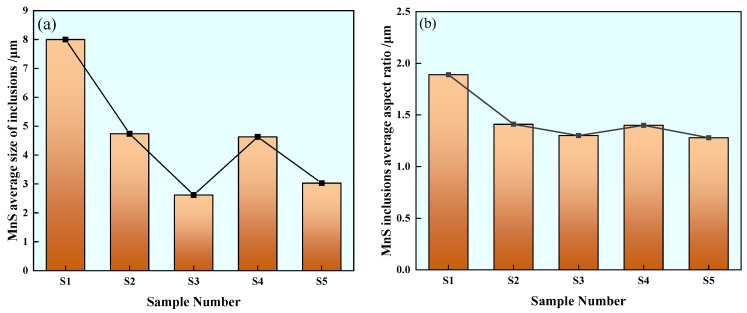
Average size and average aspect ratio of MnS inclusions: (**a**) average size and (**b**) average aspect ratio.

Among all kinds of inclusions in U75V heavy rail steel, MnS inclusions have the greatest impact on the continuity, strength, toughness and mechanical properties of the steel matrix during steel processing, so the average size and aspect ratio changes of MnS inclusions are analyzed separately. The statistics of average size changes of MnS inclusions in different samples are shown in Figure 12a. The average size of MnS inclusions in Sample S1 is 8 μm. The average size of inclusions in Sample S2 is 4.74 μm, which is about 41% lower than that in Sample S1. The average inclusion size of Sample S3 is 2.62 μm, which is about 67% lower than that of Sample S1. The average inclusion size of Sample S4 is 4.63 μm, which is about 42% lower than that of Sample S1. The average inclusion size of Sample S5 is reduced by about 62%. Compared with the MnS inclusion size of U75V steel without rare earth treatment, it is found that the MnS inclusion size in steel is greatly reduced by different rare earth contents.

The statistical variation of the average aspect ratio of MnS inclusions in different test steels is shown in Figure 12b. The average aspect ratio of MnS inclusions in Sample S1 is 1.89. The average aspect ratios of inclusions in S2, S3, S4 and S5 samples are 1.41, 1.3, 1.4 and 1.28, respectively. Compared with Sample S1, it is found that the aspect ratio of MnS inclusions decreases in the range of 25–32% after the addition of rare earth Ce to steel, and the decrease effect of MnS inclusions in Sample S5 containing 0.0042% rare earth Ce is the most obvious.

### 3.4. Effect of Rare Earth on Microstructure in U75V Steel

#### 3.4.1. Effect of Rare Earth Ce on Grain Size and Pearlite Lamellae Spacing of U75V Steel

**Figure 13:**
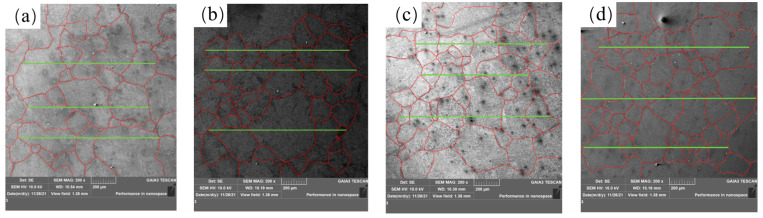
U75V steel grain size: (**a**) S1, (**b**) S2, (**c**) S4 and (**d**) S5.

Scanning electron microscopy was used to observe the macro structures in different samples after heating to 1200 °C for 1 h and water cooling, as shown in Figure 13. It can be seen from Figure 13a–d that the grain size of the steel after adding rare earth decreases.

**Figure 14:**
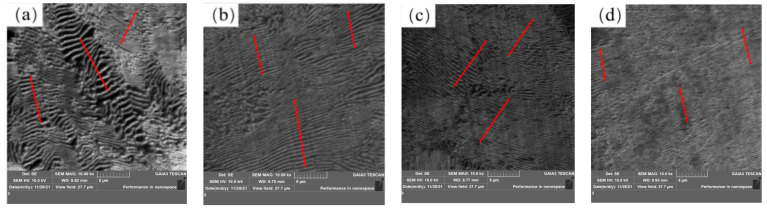
U75V steel pearlite lamellar spacing: (**a**) S1, (**b**) S2, (**c**) S4 and (**d**) S5.

**Figure 15:**
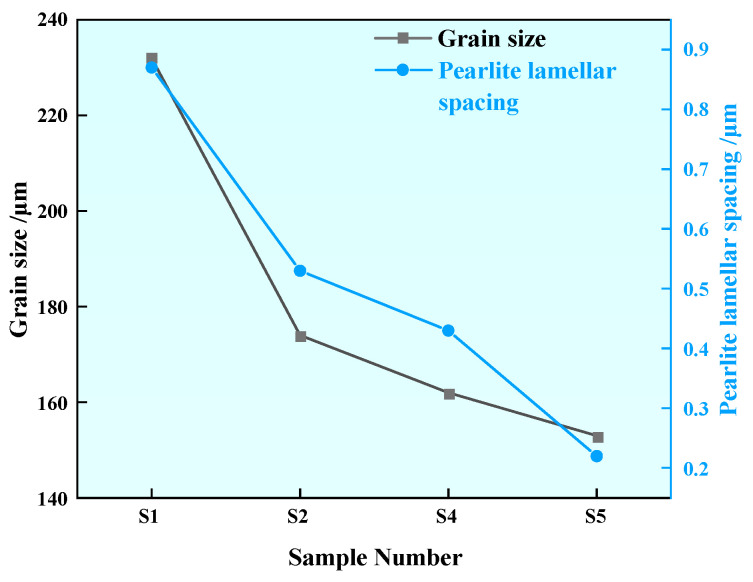
Average grain size and average pearlite lamellae spacing statistics of U75V steel.

The pearlite structure was observed by emission scanning electron microscopy, as shown in Figure 14. From Figure 14a–d, it can be seen that the layer is clearly refined after the addition of rare earth. In order to further represent the changes of grain size and pearlite lamellae spacing, Image-J 1.53a software was used to measure the grain size and pearlite lamellar layer. Eight fields of view were selected for each statistical sample, and the average value was obtained after measurement by the trans-sectional method. The measurement results are shown in Figure 15. The grain size of Sample S1 without rare earth Ce is 232 µm. With the increase in rare earth content in experimental steel, the grain sizes of Samples S2, S4 and S5 decrease to 174 µm, 162 µm and 153 µm, respectively. The grain size of Sample S5 containing 0.0042% rare earth Ce is the smallest. Compared with Sample S1 without rare earth Ce, it is reduced by about 35%. Due to the active chemical properties of Ce, the inclusion containing Ce formed after rare earth was added to the steel can act as a heterogeneous nucleating agent, which can inhibit the grain growth and optimize the structure of the steel. After the addition of rare earth Ce in the steel, the grains are continuously refined, which means that there are more grain boundaries, which can absorb more impact or tensile energy, slow down the expansion of steel cracks and improve the mechanical properties of steel [33]. The pearlite lamellae spacing of Sample S1 without rare earth Ce was 0.87 µm, and the pearlite lamellae spacings of S2, S4 and S5 samples after rare earth Ce was added were reduced to 0.53 µm, 0.43 µm and 0.22 µm, respectively, among which the pearlite lamellae spacing of Sample S5 containing 0.0042% rare earth Ce was the smallest. Compared with the Sample S1 without Ce, it was reduced by about 75%. It can be seen that Ce has an obvious thinning effect on grain size and pearlite lamellae spacing.

#### 3.4.2. Effect of Rare Earth Ce on Grain Boundary Precipitates of Experimental Steel

**Figure 16:**
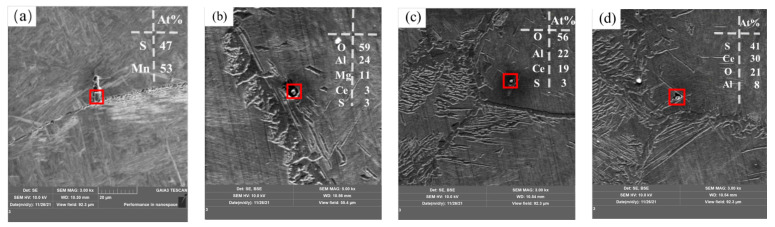
Grain boundary precipitates: (**a**) MnS, (**b**) Mg-Al-O-S-CeAlO_3_ and (**c**,**d**) CeAlO_3_-Ce_2_O_2_S.

The purification of grain boundaries and the form and distribution of rare earth inclusions in steel are important factors affecting the mechanical properties of steel. Therefore, scanning electron microscopy was used to observe precipitates near the grain boundaries, and the results are shown in Figure 16. Most of the precipitates from Sample S1 are MnS precipitates, as shown in Figure 16a. After the addition of rare earth, the composite precipitates containing Al-Mg-O-S-CeAlO_3_ and CeAlO_3_-Ce_2_O_2_S were precipitated near the grain boundary, as shown in Figure 16b–d. The consumption of S elements in liquid steel by rare earth Ce effectively reduces the segregation of MnS precipitates at the grain boundary, which is conducive to the improvement of steel properties.

### 3.5. Effect of Rare Earth Ce on Mechanical Properties of U75V Steel

#### 3.5.1. Impact Properties

**Figure 17:**
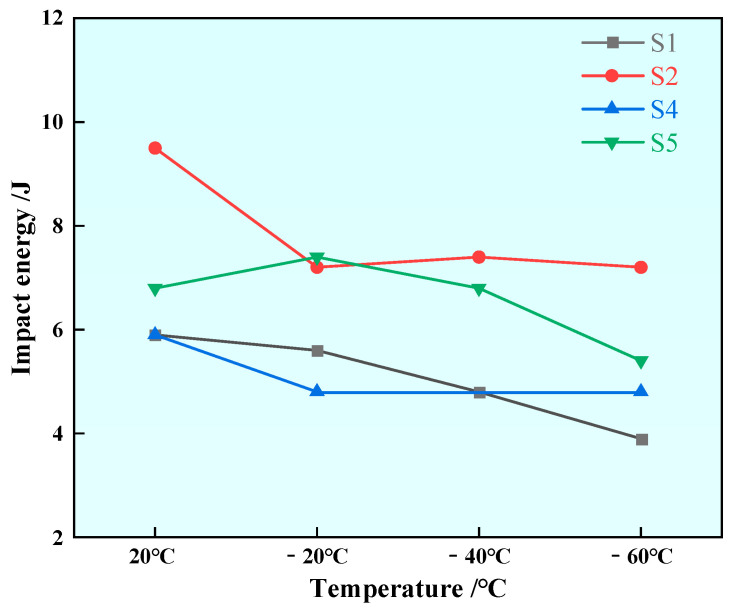
Impact energy of U75V steel at different temperatures.

Impact experiments were conducted on samples with different rare earth contents, and the results are shown in Figure 17. The impact energy of Sample S1 without rare earth elements was 5.9 J at 20 °C; the impact energies of Samples S2, S4 and S5 with rare earth element additives were 9.5 J, 5.9 J and 6.8 J at 20 °C, respectively; and the impact energy of Samples S2 and S5 at room temperature was improved. The impact energy of Sample S1 is 5.6 J at −20 °C, and the impact energies of Samples S2, S4 and S5 with rare earth additive were 7.2 J, 4.8 J and 6.8 J at −20 °C, respectively. The impact energy of Sample S1 was 4.8 J at −40 °C, and the impact energy of Sample S2 and S5 increased to 7.4 J at −40 °C after the addition of rare earth. The impact energy of Sample S1 was 3.9 J at −60 °C, and the impact energy of Samples S2, S4 and S5 increased to 7.2 J, 4.8 J and 5.4 J at −60 °C after the addition of rare earth Ce. As can be seen from Figure 17, the addition of rare earth can improve the impact performance of U75V steel, especially in different degrees under low-temperature conditions, which has good reference significance for railway construction in extremely cold areas.

**Figure 18:**
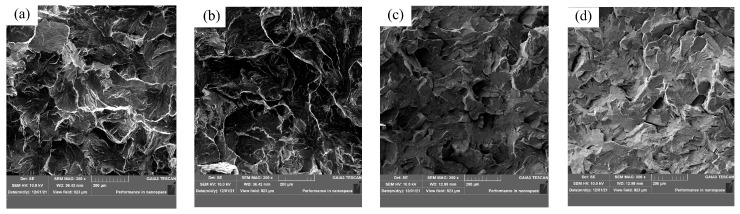
Impact fracture morphology: (**a**,**b**) without rare earths and (**c**,**d**) with rare earths.

In order to analyze the reasons for the improvement of impact performance, we observed and analyzed the fracture morphology and the inclusion from the impact fracture. Scanning electron microscopy was used to observe the fracture morphology, as shown in Figure 18. From the fracture morphology, it can be found that the fracture morphology of Sample S1 without rare earth Ce appears to be step and river, and the tear edge is large, which is a typical brittle fracture, as shown in Figure 18a,b. After the addition of rare earth Ce, the fracture morphology of the impact sample is stepped, but the fault layer tends to be gradual, the tear edge decreases and the presence of small dimples is observed, as shown in Figure 18c,d. This shows that the impact characteristics of steel change from brittle fracture to ductile fracture with the increase in rare earth Ce content in steel.

**Figure 19:**
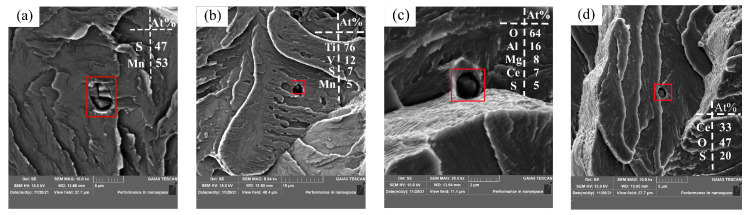
Inclusion at impact fracture: (**a**,**b**) without rare earths and (**c**,**d**) with rare earths.

Figure 19 shows the analysis of inclusions at the impact fracture. The inclusions in the impact fracture of Sample S1 without rare earth Ce are mostly MnS inclusions or MnS-Ti-V composite inclusions, as shown in Figure 19a,b. Because MnS inclusions are plastic inclusions, the deformation is large when subjected to external force, and the mechanical properties of the steel are easily reduced seriously. However, the inclusions containing (V,Ti) have high hardness, and it is difficult to change the appearance of inclusions with the change of the steel matrix, which easily produces stress concentration of the steel during use, causing the steel to produce micro-cracks. Therefore, the analysis shows that the existence of these two types of inclusions causes the impact performance of steel to decrease to a large extent. As shown in Figure 19c,d, the inclusion in the fracture of the sample after the addition of rare earth is transformed from MnS and MnS-Ti-V inclusions to inclusions containing rare earth Ce, and rare earth elements play the role of modified inclusions. Because rare earth inclusions are not easily deformed compared with MnS inclusions, and the thermal expansion coefficient of rare earth inclusions is similar to that of the steel matrix, rare earth inclusions can reduce the stress concentration caused by impact load and delay the initiation and expansion of cracks during the impact process when cracks expand to inclusions, thereby effectively improving the impact performance of steel at normal and low temperatures [34].

#### 3.5.2. Tensile Properties

**Figure 20:**
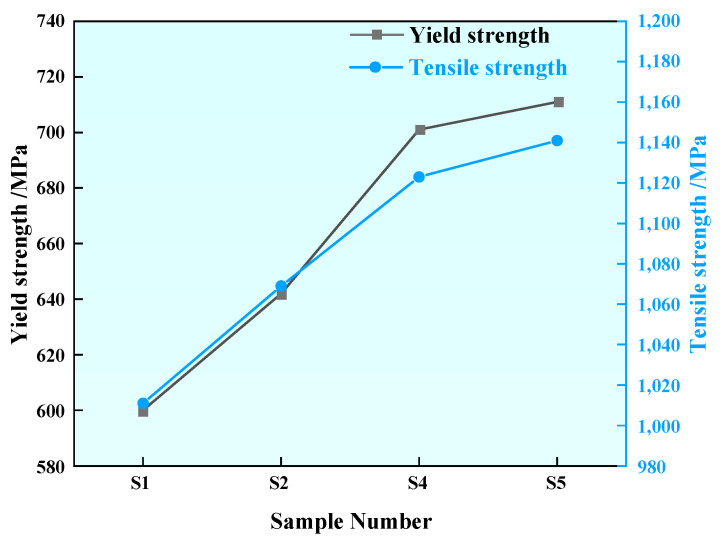
Yield strength and tensile strength of U75V steel.

Figure 20 shows the yield strength and tensile strength of the U75V steel sample before and after adding rare earth. The yield strength of Sample S1 was 600 MPa, and the yield strength of Sample S2 after adding 0.0008% rare earth Ce was 642 MPa, which is about 7% higher than that of Sample S1. The yield strength of Sample S4 after adding 0.0032% Ce was 701 MPa, which is about 16.8% higher than that of Sample S1. The yield strength of Sample S5 steel after adding 0.0042% rare earth Ce was 711 MPa, which is about 18.5% higher than that of Sample S1.

The tensile strength of Sample S1 was 1011 MPa, and the tensile strength of Sample S2 after adding 0.0008% rare earth Ce was 1069 MPa, which is about 5.7% higher than that of Sample S1. The tensile strength of Sample S4 after adding 0.0032% rare earth Ce was 1123 MPa, which is about 11.1% higher than that of Sample S1. The tensile strength of Sample S5 after adding 0.0042% rare earth Ce was 1141 MPa, which is about 12.9% higher than that of S1. The analysis of the experimental results shows that with the increasing of rare earth content within 0.0042%, both the yield strength and tensile strength of U75V steel are increased correspondingly.

**Figure 21:**
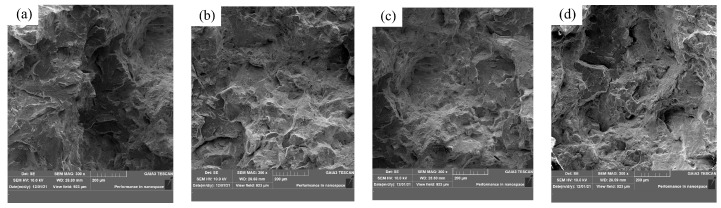
Tensile fracture morphology: (**a**) without rare earths and (**b**–**d**) with rare earths.

Scanning electron microscopy was used to observe the morphology of the tensile specimen fracture and analyze the inclusion, and the tensile fractures are shown in Figure 21. The tensile fracture morphology of Sample S1 without rare earth Ce is step and river, which is a typical brittle fracture, as shown in Figure 21a. After the addition of different rare earth contents, obvious dimples were generated in the tensile fracture of the sample, and the tear edges became smaller, as shown in Figure 21b–d. This shows that the change from brittle fracture to ductile fracture is beneficial to the improvement of tensile properties, which is consistent with the experimental results.

**Figure 22:**
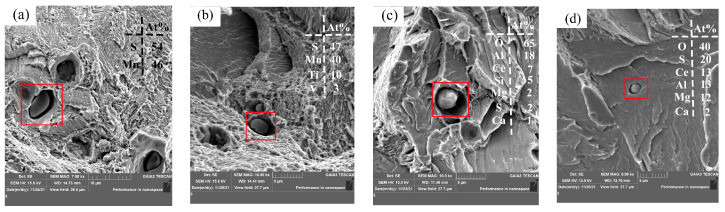
Inclusion at tensile fracture: (**a**,**b**) without rare earths and (**c**,**d**) with rare earths.

Figure 22 shows the analysis of inclusions in the fracture of the tensile sample. The results show that the inclusions of Sample S1 without rare earth Ce are mostly MnS inclusions and complex inclusions formed by MnS, Ti and V, as shown in Figure 22a,b. The inclusion in the tensile fracture after the addition of rare earth Ce was modified into a complex inclusion containing rare earth Ce, as shown in Figure 22c,d. It has been suggested that inclusions are an important factor affecting the mechanical properties of steel. Rare earth inclusion is generally not the cause of crack growth, but sulfide is the direct cause of fatigue crack growth. This is because when the crack extends to the inclusion, the rare earth inclusion slows down the stress concentration, thereby preventing the crack growth [28,35]. Rare-earth inclusions have better fusion with the steel matrix than MnS inclusions and MnS-Ti-V complex inclusions. Rare-earth inclusions absorb a large amount of stress concentration before deformation and fracture of the tensile specimen, which causes the crack propagation of the tensile specimen under external loads to be slow [36].

## 4. Conclusions

In this paper, the thermodynamic calculation of Ce-containing inclusions is first carried out to study the precipitation of Ce-containing inclusions in liquid steel. In the experimental part, the changes in the appearance, type and size of inclusions in U75V heavy rail steel before and after the addition of rare earth Ce are studied, and the macro-structure and pearlite lamination of U75V steel are also observed and analyzed. Finally, the influence of rare earth Ce on the mechanical properties of U75V steel is studied, and the research results are as follows:

1. According to the thermodynamic calculation results of the Wagner model, the value is 0.1 < O/S < 0.2; under the condition of a certain Al content, CeAlO_3_ inclusion is first precipitated in U75V steel at 1600 °C when the Ce content is less than 0.0075%, and Ce_2_O_2_S inclusion is first precipitated in liquid steel when the Ce content is greater than 0.0075%. The precipitation stability of Ce inclusions is not affected by the decrease in temperature, but the formation of CeO_2_, Ce_2_O_3_ and CeAlO_3_ inclusions is inhibited by the increase in rare earth Ce.
2. U75V steel without rare earth Ce contains irregular MnS and complex inclusions containing Al, Si, Ca, Mn, S, O, etc. After the addition of rare earth Ce, the pure MnS in the steel decreases, and rare earth inclusions such as CeAlO_3_ and Ce_2_O_2_S and other complex inclusions containing Al, Si, Ca, Mn, S, O and other elements appear. With the increase in Ce content, the sizes and aspect ratios of inclusions in the steel are reduced to some extent, and the addition of rare earth Ce has a certain thinning effect on pure MnS inclusions.
3. Rare earth Ce can effectively refine the grain size of U75V steel and significantly reduce the pearlite lamellae spacing. The analysis of precipitates near the grain boundary shows that rare earth elements can reduce the segregation of sulfide at the grain boundary.
4. The addition of rare earth Ce can improve the normal- and low-temperature impact performance of U75V steel; in particular, the improvement effect of low-temperature impact performance is obvious; this has certain practical significance in the application of rails in extremely cold weather. The experiment also found that with the increase in Ce content, the yield strength and tensile strength of U75V steel also increased.

## Figures and Tables

**Table 1:** Chemical composition of raw materials used for smelting U75V heavy rail steel (mass fraction%).

Calcined Petroleum Coke	Ferrosilicon	Ferromanganese	Ferrovanadium	Ferrocerium	Pure Iron
[%C] = 98.5 [%Volatiles] = 1.5	[%Si] = 72.53	[%Mn] = 97.15	[%V] = 46	[%Ce] = 30 [%Fe] = 70 [%C] ≤ 0.01	[%Fe] = 99.99
	[%C] = 0.12	[%C] = 0.07	[%C] = 0.17		
	[%P] = 0.03	[%P] = 0.04	[%P] = 0.06		
	[%S] = 0.02	[%S] = 0.04	[%S] = 0.01		
	[%Fe] = 27.3	[%Fe] = 2.7	[%Fe] = 53.76		

**Table 2:** Chemical compositions of experiment steels (mass fraction%).

Sample Numbers	C	Si	Mn	V	S	P	O	Ce
S1	0.798	0.660	0.980	0.061	0.013	<0.005	0.00365	0
S2	0.791	0.716	1.009	0.0068	0.01	0.037		0.0008
S3	0.832	0.713	0.978	0.062	0.011	0.003	0.00331	0.0013
S4	0.785	0.673	1.017	0.069	0.012	0.007		0.0032
S5	0.831	0.709	0.913	0.046	0.007	0.006		0.0042

**Table 3:** U75V steel composition for calculation.

C	Si	Mn	V	S	P	Ca	Al	O	Ce
0.75	0.6	0.8	0.06	0.02	0.02	0.001	0.001	0.003	0.0025
0.75	0.6	0.8	0.06	0.02	0.02	0.001	0.001	0.0028	0.006
0.75	0.6	0.8	0.06	0.02	0.02	0.001	0.001	0.0026	0.008
0.75	0.6	0.8	0.06	0.02	0.02	0.001	0.001	0.0024	0.012
0.75	0.6	0.8	0.06	0.02	0.02	0.001	0.001	0.002	0.014

**Table 4:** Gibbs free energy of rare earth inclusions generated by reaction in liquid steel [27,28].

Reaction	$\Delta G^{\theta }/({J\;\mathrm{mol}}^{-1})$
$\left[Ce]+3/2[O]=1/2Ce_{2}O_{3}(s\right)$	−715560+180T
$\left[Ce]+2[O]=CeO_{2}(s\right)$	−852720+249.96T
[Ce]+[S]=CeS(s)	−422100+120.38T
$\left[Ce]+3/2[S]=1/2Ce_{2}S_{3}(s\right)$	−536420+163.86T
$\left[Ce]+4/3[S]=1/3Ce_{3}S_{4}(s\right)$	−497670+146.3T
$\left[Ce]+[O]+1/2[S]=1/2Ce_{2}O_{2}S(s\right)$	−675700+165.5T
$\left[Ce]+[Al]+3[O]=CeAlO_{3}(s\right)$	−1366460+364T

**Table 5:** Interaction coefficient $e_{i}^{j}$ of various elements in liquid steel at 1600 °C [20,27,29].

	C	Si	Mn	V	S	P	Ca	Al	O	Ce
O	−0.45	−0.013	−0.021	−0.3	−0.133	0.07	−313	−3.85	−0.02	−12.1
Al	0.091	0.0056	−0.02	0.025	0.03	0.033	−0.047	0.045	−6.6	−0.43
Ce	0.091	-	-	−0.33	1.77	1.77	-	−2.25	−106	−0.006

**Table 6:** Precipitation and evolution sequence of rare earth inclusions at 1600 °C.

Al	Ce	Precipitation Sequence of Ce-Containing Inclusions
0.0010%	0.0025–0.0058%	CeAlO_3_ > Ce_2_O_2_S > Ce_2_O_3_ > CeO_2_ > Ce_3_S_4_ > Ce_2_S_3_ > CeS
	0.0058–0.0075%	CeAlO_3_ > Ce_2_O_2_S > Ce_2_O_3_ > Ce_3_S_4_ > CeO_2_ > Ce_2_S_3_ > CeS
	0.0075–0.0078%	Ce_2_O_2_S > CeAlO_3_ > Ce_2_O_3_ > Ce_3_S_4_ > CeO_2_ > Ce_2_S_3_ > CeS
	0.0078–0.0102%	Ce_2_O_2_S > CeAlO_3_ > Ce_2_O_3_ > Ce_3_S_4_ > Ce_2_S_3_ > CeO_2_ > CeS
	0.0102–0.014%	Ce_2_O_2_S > CeAlO_3_ > Ce_2_O_3_ > Ce_3_S_4_ > Ce_2_S_3_ > CeS > CeO_2_

## Data Availability

Data are contained within the article.
